# Development and validation of a prediction model for unexpected poor ovarian response during IVF/ICSI

**DOI:** 10.3389/fendo.2024.1340329

**Published:** 2024-03-04

**Authors:** Xiaohang Xu, Xue Wang, Yilin Jiang, Haoyue Sun, Yuanhui Chen, Cuilian Zhang

**Affiliations:** ^1^ Reproductive Medical Center, People’s Hospital of Zhengzhou University, Zhengzhou, China; ^2^ Reproductive Medical Center, Henan Provincial People’s Hospital, Zhengzhou, China

**Keywords:** predictive model, nomogram, poor ovarian response, ovarian reserve, IVF/ICSI

## Abstract

**Background:**

Identifying poor ovarian response (POR) among patients with good ovarian reserve poses a significant challenge within reproductive medicine. Currently, there is a lack of published data on the potential risk factors that could predict the occurrence of unexpected POR. The objective of this study was to develop a predictive model to assess the individual probability of unexpected POR during *in vitro* fertilization/intracytoplasmic sperm injection (IVF/ICSI) treatments.

**Methods:**

The development of the nomogram involved a cohort of 10,404 patients with normal ovarian reserve [age, ≤40 years; antral follicle count (AFC), ≥5; and anti-Müllerian hormone (AMH), ≥1.2 ng/ml] from January 2019 to December 2022. Univariate regression analyses and least absolute shrinkage and selection operator regression analysis were employed to ascertain the characteristics associated with POR. Subsequently, the selected variables were utilized to construct the nomogram.

**Results:**

The predictors included in our model were body mass index, basal follicle-stimulating hormone, AMH, AFC, homeostasis model assessment of insulin resistance (HOMA-IR), protocol, and initial dose of gonadotropin. The area under the receiver operating characteristic curve (AUC) was 0.753 [95% confidence interval (CI) = 0.7257–0.7735]. The AUC, along with the Hosmer–Lemeshow test (*p* = 0.167), demonstrated a satisfactory level of congruence and discrimination ability of the developed model.

**Conclusion:**

The nomogram can anticipate the probability of unexpected POR in IVF/ICSI treatment, thereby assisting professionals in making appropriate clinical judgments and in helping patients to effectively manage expectations.

## 1 Introduction

During treatment of infertile couples, controlled ovarian stimulation (COS) can facilitate the synchronous growth of numerous follicles and oocyte maturation, thereby enhancing the oocyte yield, which plays a pivotal role in achieving *in vitro* fertilization–embryo transfer (IVF-ET) success. However, despite appropriate ovarian stimulation, some patients may still experience suboptimal outcomes with lower than anticipated numbers of retrieved oocytes. This condition is referred to as “poor ovarian response” (POR) (1).

The occurrence of POR ranges from 9% and 24% in the COS procedure (2), resulting in a decrease in ovarian response, a lower number of retrieved oocytes, a reduced rate of pregnancy, and an elevated likelihood of cycle cancellation and miscarriage (3). How to identify and manage the POR population has been a key area in reproductive medicine. The Bologna criteria, established by the European Society of Human Reproduction and Embryology (ESHRE), are universally acknowledged standards for POR (4). Populations defined as POR with distinct clinical features and prognoses are categorized together. Later, the “Patient-Oriented Strategies Encompassing Individualized Oocyte Number” (POSEIDON) criteria were proposed as novel low-prognosis categories for individuals with POR in 2016 (5). The POSEIDON criteria divide poor responders into four groups based on ovarian reserve markers, i.e., age, anti-Müllerian hormone (AMH), and antral follicle count (AFC), to better assess patients. Poor responders are further distributed into the “expected POR group” and the “unexpected POR group” according to POR heterogeneity. Both of these key criteria defined that a retrieval oocyte count ≤3 is POR. In existing studies, the identification of the POR population mainly relies on ovarian reserve markers. However, these biomarkers are suboptimal for predicting patients who are hyporesponders (6). Despite a good ovarian reserve function, a subset of patients may still exhibit POR. In the clinical setting, there is a scarcity of precise methods for predicting unexpected POR. The factors influencing POR occurrence are intricate and diverse. Factors such as body mass index (BMI), gonadotropin (Gn) dose, COS protocols, and insulin metabolism could also have an influence on the ovarian response. Hence, the aim of this study was to identify the factors that can predict unforeseen POR and develop a personalized prediction model in order to identify and implement individualized strategies for patients with unexpected POR. The prediction model was demonstrated as a nomogram that can be applied during patient counseling and clinical decision-making.

## 2 Materials and methods

### 2.1 Subjects

**Figure 1:**
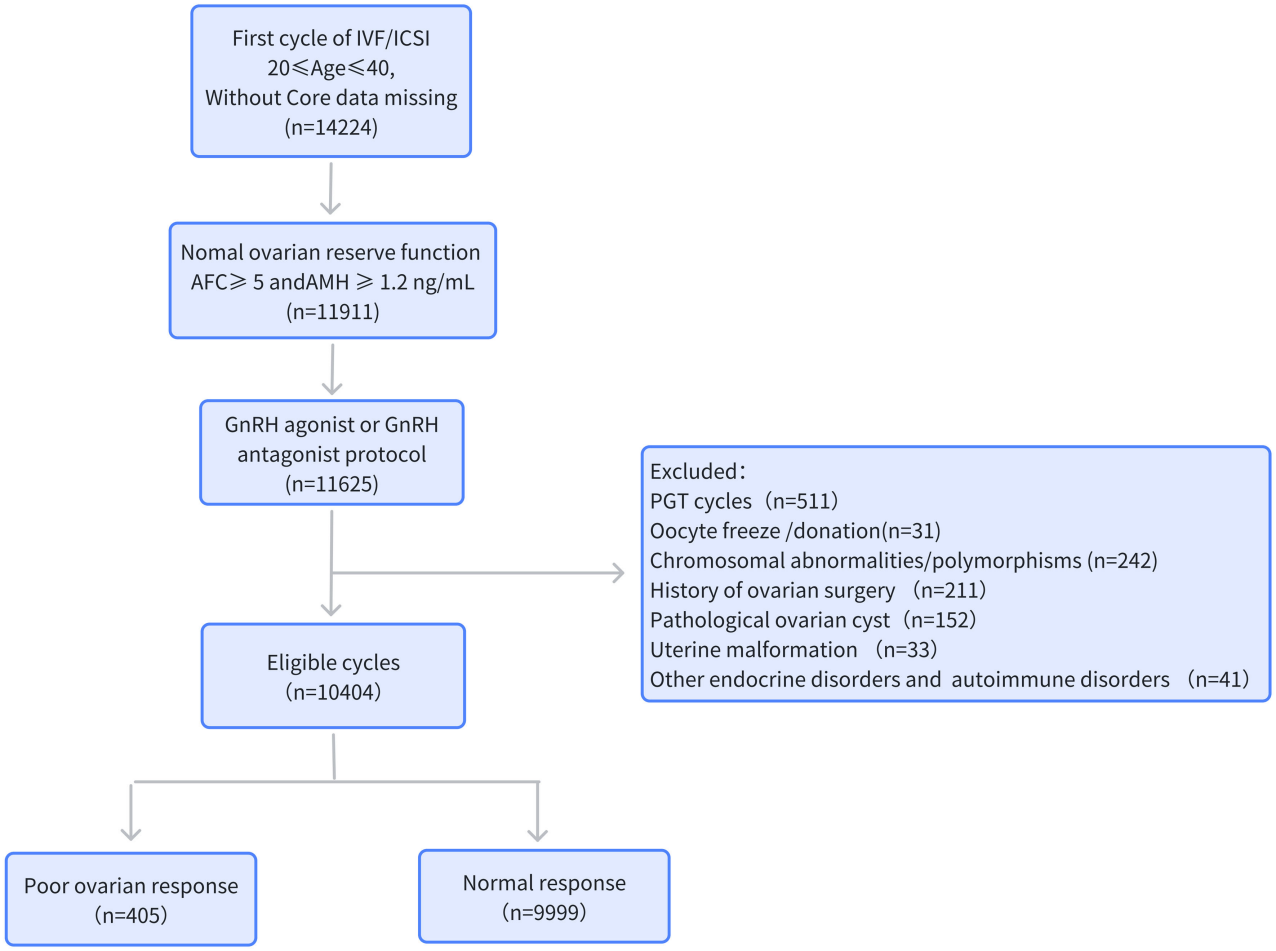
Flowchart of the data collection process. *AMH*, anti-Müllerian hormone; *AFC*, antral follicle count; *BMI*, body mass index; *PGT*, pre-implantation genetic testing.

A retrospective analysis was conducted in patients who underwent IVF or intracytoplasmic sperm injection (ICSI) treatment in Henan Provincial People’s Hospital between January 1, 2019, and December 31, 2022. The criteria for inclusion were as follows: 1) first cycle of IVF/ICSI treatment; 2) 20 years ≤ age ≤ 40 years; 3) normal ovarian reserve function: AFC ≥ 5 and AMH ≥ 1.2 ng/ml; 4) gonadotropin-releasing hormone (GnRH) agonist or GnRH antagonist protocols; and 5) complete relevant records in the patient electronic medical record system. The exclusion criteria were the following: 1) pre-implantation genetic testing (PGT) cycle; 2) cycles involving oocyte cryopreservation or donation; 3) female chromosomal abnormalities or chromosomal polymorphisms; 4) coexisting factors that may affect ovarian response, such as history of ovarian surgery or pathological ovarian cyst; 5) uterine abnormalities; and 6) the following metabolic disorders: diabetes, hypertension, hyperprolactinemia, hyperthyroidism, hypothyroidism, and autoimmune disorders. The process for the inclusion and exclusion of patients is presented in **Figure 1**.

According to the Bologna and the POSEIDON criteria, in this study, cases with no more than 3 were established as “POR.” All patients were categorized into two groups based on their ovarian response: the “unexpected poor ovarian response” group (POR group) and the “normal ovarian response” group (NOR group).

### 2.2 Data acquisition

The primary data for this study were derived from the electronic medical record system of our hospital, encompassing all assisted reproductive data. This analysis was authorized by the Henan Provincial People’s Hospital Ethics Committee (no. 2022139).

The basal sex hormone and AMH levels were measured on days 2–4 of the menstrual cycle in patients undergoing IVF/ICSI. The concentration of AMH was determined using the electrochemiluminescence immunoassay method [sensitivity = 0.1 ng/ml, coefficient of variation (CV) < 3.2%] on a fully automatic chemiluminescence immunoassay analyzer (Cobas8000 e602; Roche Diagnostics GmbH, Mannheim, Germany) in the laboratory of the Department of Reproductive Endocrinology at Henan Provincial People’s Hospital. Our laboratory undergoes annual qualification checks by the External Quality Assessment of Clinical Laboratory Center annually (Ministry of Health of the People’s Republic of China, Beijing, China). The assay characteristics were as follows: sensitivity = 0.16 ng/ml, intra-assay CV = 3.2%, and inter-assay CV = 7.0%. AFC was defined as the sum of the number of follicles 2–10 mm in diameter in both ovaries. Assessment was performed using transvaginal sonography by a relatively fixed group of 3 experienced reproductive physicians before COS. These doctors underwent standardized training and adhered to uniform standards. Transvaginal ultrasonography (Voluson E8 Expert; GE Healthcare, Chicago, IL, USA) was performed using a 4- to 9-MHz probe. Homeostasis model assessment of insulin resistance (HOMA-IR) was calculated using the following formula: HOMA-IR = fasting blood glucose (mmol/L) × fasting insulin (μU/ml)/22.5 (7). Pregnancy outcome data were collected until September 2023. Live birth rate referred to a live birth per embryo transfer (ET) cycle. Patients who were more than 3 months pregnant but had not yet delivered were not included in the calculation. The cumulative pregnancy rate was defined as the total number of pregnancies achieved across all ET cycles in one retrieval cycle.

### 2.3 Ovarian stimulation protocol, embryo transfer, and luteal support

The COS protocol and Gn dose were customized based on the patient’s age, weight, and ovarian reserve using a step-up regimen for the Gn dose. The protocols included the early follicular-phase long-acting GnRH agonist long protocol, the mid-luteal-phase short-acting GnRH agonist long protocol, and the antagonist flexible protocol. In the short-acting GnRH agonist protocol, the patients were injected 0.1 mg triptorelin from day 6 to day 8 after ovulation for 14–18 days until achieving pituitary downregulation. Thereafter, Gn and 0.05 mg/day triptorelin were administered together until human chorionic gonadotropin (hCG) triggering was scheduled. Triptorelin acetate (triptorelin, 3.75 mg) was given during the menstrual period in the long-acting GnRH agonist regimen. When satisfactory pituitary downregulation was achieved after approximately 30–35 days, Gn was injected every day. In the GnRH antagonist flexible protocol, Gn was administrated from day 2 to day 3 of this menstrual cycle and a GnRH antagonist (cetrotide, 0.25 mg) injected daily from day 6 to day 7 of stimulation when one dominant follicle was ≥12 mm. When one dominant follicle was ≥20 mm in diameter or three follicles were ≥17 mm in diameter or more than 2/3 follicles were ≥16 mm in diameter, recombinant hCG (250 μg) or urinary hCG (2,000 U) was injected. Transvaginal ultrasound-guided aspiration was conducted 35–38 h following hCG injection to pick up oocytes. The Istanbul Consensus scoring system was applied for embryo evaluation (8). A transferable embryo referred to an embryo with ≥ 4 cells, < 26% fragmentation, and either no asymmetry or moderate asymmetry, which could be transferred or cryopreserved. ET was conducted on day 3 or day 5 after oocyte retrieval. The endometrial preparation protocol was selected individually in frozen ET cycles. Approximately 1–2 cleavage embryos or blastocysts would be transferred. Combined vaginal and oral progesterone was administered for luteal-phase support until 8–10 weeks.

### 2.4 Statistical analyses

Data analysis was conducted using EmpowerStats statistical software (X&Y Solutions). Continuous variables were reported as the mean ± standard deviation (SD), while categorical variables were expressed as number and percentages. Univariable logistic regression analyses were performed to identify the relevant factors for unexpected POR. The least absolute shrinkage and selection operator (LASSO) binary logistic regression analyses were implemented with the “glmnet” package in R for variable selection. In addition, a nomogram was created using the “rms” package to offer graphical representations of the selected factors in the model and to facilitate users in calculating probabilities (9). For validation, bootstrap and the Hosmer–Lemeshow test were carried out to estimate the goodness-of-fit of the model. Receiver operating characteristic (ROC) curves and the areas under the curves (AUC) were calculated to compare the prediction model and single related factors. Optimal cutoff values were determined using the Youden index.

## 3 Results

### 3.1 Analysis of patients’ baseline characteristics

This study included a total of 10,404 patients, 405 cases (3.89%) in the unexpected POR group and 9,999 (96.11%) cases in the NOR group. A total of 13 cycles were canceled after oocyte retrieval and 5,850 cycles underwent fresh ET. **Table 1** outlines the baseline characteristics. Significant differences were observed in age, weight, BMI, basal follicle-stimulating hormone (bFSH), basal progesterone, AMH, AFC, fasting blood glucose, fasting insulin, HOMA-IR, protocol composition, Gn initial dose, Gn duration, and total Gn usage between the two groups (*p* < 0.05). No statistical significance was observed in the infertility type, infertility duration, polycystic ovary syndrome (PCOS) composition, basal luteinizing hormone (LH) level, estrogen (E_2_), prolactin (PRL), testosterone (T), and progesterone (P) (*p* > 0.05).

**Table 1:** Comparison of the basic parameters of the study population using univariable logistic regression analysis.

Parameter	POR group	NOR group	*p*-value
*N*	405	9,999	
Age (years)	31.60 ± 4.22	30.36 ± 4.01	<0.001
Weight	64.79 ± 12.17	60.72 ± 10.08	<0.001
BMI	24.90 ± 4.20	23.33 ± 3.69	<0.001
Infertility type			0.178
Primary	202 (49.88%)	5,328 (53.29%)	
Secondary	203 (50.12%)	4,671 (46.71%)	
Infertility years	3.74 ± 2.92	3.53 ± 2.56	0.097
PCOS			0.0394
Yes	54 (13.33%)	1,728 (17.29%)	
No	351 (86.67%)	8,271 (82.71%)	
Basal FSH	6.86 ± 2.11	6.28 ± 1.71	<0.001
Basal LH	5.44 ± 3.06	5.89 ± 3.46	0.011
Basal PRL	16.03 ± 7.99	17.31 ± 9.00	0.010
Basal E_2_	39.13 ± 18.28	40.32 ± 17.70	0.193
Basal T	0.32 ± 0.35	0.35 ± 1.04	0.647
Basal P	0.29 ± 0.25	0.32 ± 0.24	0.006
AMH	3.49 ± 2.63	4.67 ± 3.28	<0.001
AFC	13.79 ± 6.68	16.08 ± 5.89	<0.001
Glucose	5.01 ± 0.56	4.86 ± 0.52	<0.001
Insulin	14.73 ± 8.36	12.03 ± 7.35	<0.001
HOMA-IR	3.33 ± 2.05	2.64 ± 1.73	<0.001
Protocol			<0.001
GnRh antagonist	174 (42.96%)	1,853 (18.53%)	
GnRh agonist	231 (57.04%)	8,146 (81.47%)	
Initial dose of Gn	189.38 ± 63.16	153.61 ± 47.63	<0.001
Gn duration	11.16 ± 2.69	10.45 ± 3.64	<0.001
Total Gn	2,182.91 ± 921.06	2,421.36 ± 1,211.21	<0.001

Values are the mean ± standard deviation or number (percentage).

*POR*, poor ovarian response; *NOR*, normal ovarian response; *PCOS*, polycystic ovary syndrome; *BMI*, body mass index; *FSH*, follicle-stimulating hormone; *LH*, luteinizing hormone; *PRL*, prolactin; *E*_2_, estrogen; *T*, testosterone; *P*, progesterone; *AFC*, total antral follicle count; *HOMA-IR*, homeostasis model assessment of insulin resistance; *Gn*, gonadotropin; *CI*, confidence interval.

### 3.2 Analysis of patients’ laboratory index and clinical outcomes

The NOR group exhibited significantly higher numbers of dominant follicles on hCG trigger day, retrieved oocytes, MII oocytes, 2PN embryos, and transferable embryos and higher rates of fresh cycle pregnancy and cumulative pregnancy compared to the POR group (*p* < 0.05). There was no statistically significant difference in the ectopic rate, abortion rate, and live birth rate of fresh cycle between the two groups (*p* > 0.05) (**Table 2**).

**Table 2:** Comparison of the laboratory index and clinical outcomes of the study population.

Index	POR group	NOR group	*p*-value
*N*	405	9,999	
Dominant follicles on hCG trigger day	4.18 ± 2.98	10.68 ± 5.03	<0.001
Oocyte	2.45 ± 0.63	12.68 ± 6.47	<0.001
No. of MII	2.07 ± 0.85	10.62 ± 5.76	<0.001
No. of 2PN	1.46 ± 0.93	7.58 ± 4.61	<0.001
Transferrable embryos	1.17 ± 0.89	4.51 ± 3.01	<0.001
Pregnancy outcomes			
Clinical pregnancy rate of fresh cycle	47.75% (106/222)	60.47% (3,403/5,628)	<0.001
Ectopic pregnancy rate of fresh cycle	1.89% (2/106)	1.32% (45/3,403)	0.391^b^
First-trimester miscarriage rate of fresh cycle	18.92% (19/106)	13.46% (458/3,403)	0.1865
Live birth rate of fresh cycle^a^	78.35% (76/97)	84.13% (2,668/3,171)	0.126
Cumulative pregnancy rate (per retrieval cycle)	33.33% (135/405)	73.76% (7,375/9,999)	<0.001

Values are the mean ± standard deviation or number (percentage).

*POR*, poor ovarian response; *NOR*, normal ovarian response; *hCG*, human chorionic gonadotropin.

a Patients who were more than 3 months pregnant but had not yet delivered were not included (9 patients in the POR group and 232 patients in the NOR group).

b Fisher’s exact test.

### 3.3 Feature selection and parameter building

**Figure 2:**
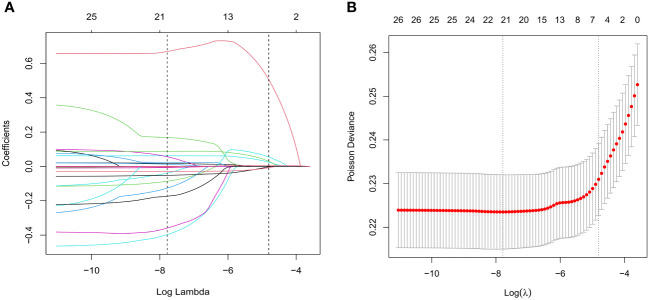
Variable selection using the least absolute shrinkage and selection operator (LASSO) regression algorithm. **(A)** Lasso regression path diagram. **(B)** LASSO coefficient profiles of the characteristics. Parameters were screened out by 10-fold cross-validation and using lambda.1se as the criteria.

Aiming to screen associated factors for unexpected POR, we adopted a LASSO regression mechanism, which is an ideal method for performing interaction testing, variable selection, and parameter estimation without overfitting. A total of 21 potential related variables (listed in **Table 1**) were analyzed. Of these, seven variables were ultimately suggested as predictors (namely, BMI, bFSH, AMH, AFC, HOMA-IR, protocol, and initial dose of Gn) for unanticipated POR using the LASSO regression model, as shown in **Figures 2A, B**.

### 3.4 Development of an individualized prediction model

**Figure 3:**
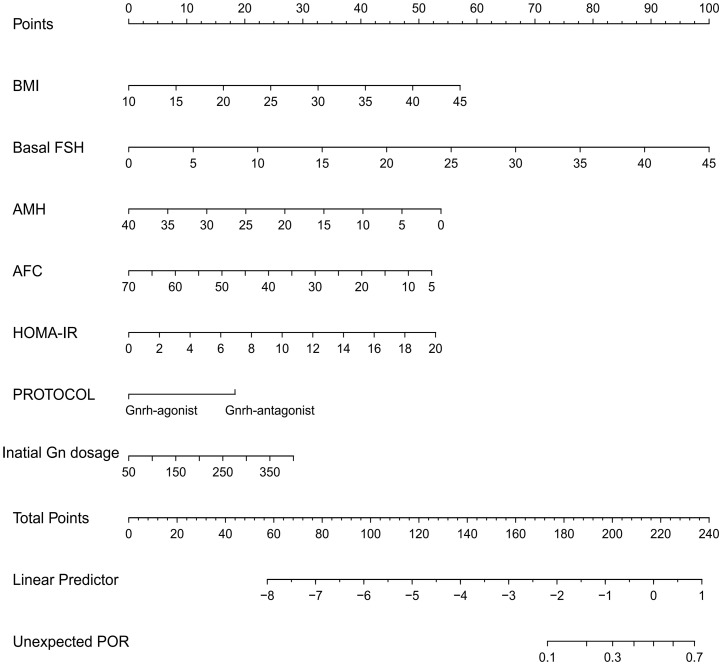
Nomogram for predicting unanticipated poor ovarian response (POR). The point of each variable was obtained at the intersection of the vertical line drawn from each variable to the point ruler. The total score of all factors was calculated and a descending line drawn from the “Total Points” until it intersected with the lower line to acquire the probability of POR.

The developed model for estimating the POR used the selected variables, i.e., BMI, bFSH, AMH, AFC, HOMA-IR, protocol, and initial dose of Gn, as indicators. The nomogram for prediction is depicted in **Figure 3**. Each parameter was assigned a vertical extension (shown in the top points bar) individually. The total score was determined by summing up the scales for each factor. The overall point projected on the bottom scale suggests the likelihood of poor response. The equation for the nomogram is as follows: logit(unexpected POR) = −4.54532 + 0.08169 × BMI + 0.11129 × bFSH − 0.06736 × AMH − 0.04022 × AFC + 0.13237 × HOMA-IR − 0.91707 × protocol + 0.00406 × initial dose of Gn.

### 3.5 Validation of the nomogram

**Figure 4:**
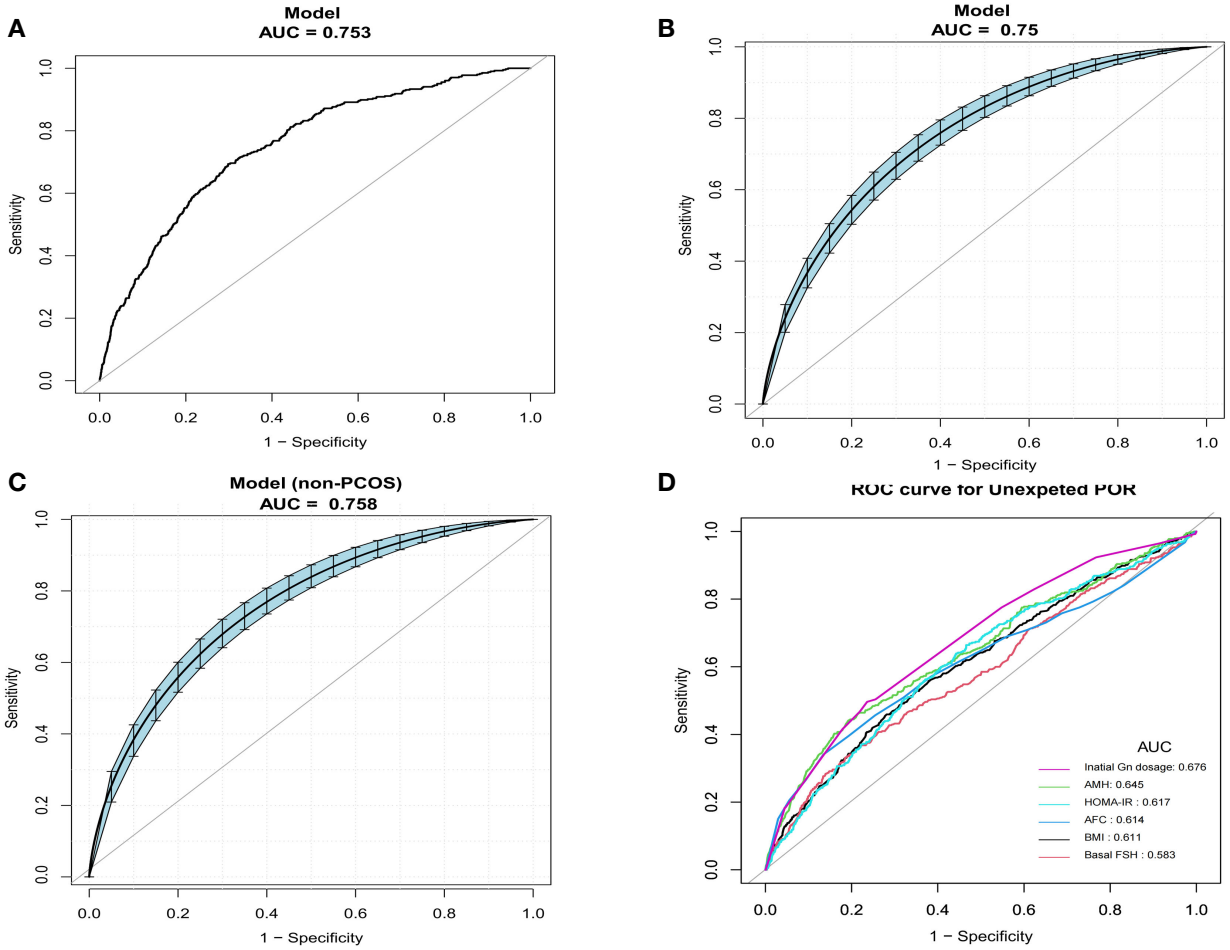
Comparison of the predictive potential of different variables for poor ovarian response (POR) using receiver operating characteristic (ROC) curves. **(A)** Predictive ability of the nomogram models for unexpected POR. **(B)** Confidence interval of the area under the curve (AUC) and significance tests using bootstrap resampling (bootstrap resampling times = 500). **(C)** Confidence interval of the AUCs in non-polycystic ovary syndrome (PCOS) patients (bootstrap resampling times = 500). **(D)** Predictive ability of individual variables.

Analyses of the ROC curves were performed to investigate the forecast value of the prediction model. The results of AUC analyses indicated that our model demonstrated good discriminative potential, with an AUC of 0.753 (95%CI = 0.730–0.77.7). The AUC confidence interval and significance test were obtained using the bootstrap method (bootstrap resampling times = 500) (**Figures 4A, B**). The Hosmer–Lemeshow test exhibited a non-significant difference (*p* = 0.167), demonstrating the satisfactory level of effectiveness and reliability of this model. Furthermore, the model was also validated in non-PCOS patients and exhibited fairly good discriminative ability, with an AUC of 0.758 (95%CI = 0.732–0.783). The Hosmer–Lemeshow test showed a non-significant difference (*p* = 0.112) (**Figure 4C**). In addition, we compared the predictive potential of the single variables and the predictive model using the ROC curves. The AUC of the nomogram was significantly higher than that of the single variables BMI, bFSH, AMH, AFC, HOMA-IR, and initial dose of Gn (**Figure 4D**, **Table 3**).

**Table 3:** Predictive accuracy of the model and various variables for unexpected POR.

Variable	AUC	95%CI	Best threshold	Specificity (%)	Sensitivity (%)	PPV (%)	NPV (%)
Unexpected POR model	0.7533	0.7257–0.7735	–	0.7018	0.6938	0.0861	0.9826
BMI	0.6111	0.5821–0.6402	23.9450	0.6213	0.5580	0.0563	0.9720
bFSH	0.5826	0.5524–0.6128	7.4050	0.8148	0.3309	0.0675	0.9678
AMH	0.6453	0.6156–0.6750	2.2050	0.8066	0.4420	0.0847	0.9727
AFC	0.6136	0.5814–0.6457	9.5000	0.8653	0.3407	0.0929	0.9701
HOMA-IR	0.6174	0.5892–0.6457	2.2887	0.5341	0.6642	0.0546	0.9752
Initial dose of Gn	0.6760	0.6493–0.7026	168.7500	0.7640	0.4963	0.0785	0.9740

*AUC*, area under the curve; *CI*, confidence interval; *NPV*, negative predictive value; *PPV*, positive predictive value; *POR*, poor ovarian response; *bFSH*, basal follicle-stimulating hormone; *AMH*, anti-Müllerian hormone; *HOMA-IR*, homeostasis model assessment of insulin resistance; *Gn*, gonadotropin.

## 4 Discussion

Precise prediction of ovarian response is essential for successful ovarian stimulation. The association of the ovarian reserve biomarkers including age, AFC, and AMH with ovarian response has been clarified (10). However, a subset of patients with a seemingly normal ovarian reserve may manifest as “unexpected poor responders” during COS. Currently, there is no consensus on how to identify these patients before oocyte retrieval and no acknowledged mathematical model for prediction. Early detection of infertile women with declined ovarian sensitivity is important as it could impact the treatment decision during ovulation induction. Therefore, our study developed and validated a predictive model for unexpected POR. To our knowledge, this is the first work on the prediction of POR in infertile patients with normal ovarian reserve.

Consistent with multiple studies, our research revealed that the ovarian reserve markers (i.e., bFSH, AMH, and AFC) can serve as predictive factors to identify individuals at risk of unanticipated POR. A large sample real-world analysis reported that AFC and AMH showed remarkable precision in predicting POR. Thresholds of AFC ≤ 5 and AMH ≤ 1.18 ng/ml were suggested. Age was also suggested for consideration (11). Xu et al. established a mathematical model with AMH, AFC, and bFSH as predictors to estimate the probability of POR. The AUCs in the inner/outer validation data were 0.861/0.850 (12). Despite AFC and AMH being ovarian reserve markers with high sensitivity, their reliability is not absolute as they could yield false-positive results at rates ranging from 10% to 20% (9). In this study, the AUC of the developed model was 0.7533, which is noticeably higher than those of the single predictors including the ovarian reserve marker, BMI, HOMA-IR, and initial dose of Gn. This finding indicates that the combination of different predictors can improve the predictive accuracy.

Our study also suggested that metabolic indicators are associated with POR. We therefore incorporated HOMA-IR and BMI into the predictive model. This is the primary innovation of our research and the main distinction from previous findings, given that patients with PCOS have a higher risk of insulin resistance and are overweight. The predictive model was also validated in non-PCOS patients and showed similar predictive performance. HOMA-IR, a commonly used metric to assess insulin sensitivity, exhibits high sensitivity and specificity in measuring insulin resistance (13). Several studies have reported that insulin resistance may directly impact oocyte maturation and ovulation in patients with PCOS (14, 15). However, only a few studies have focused on the relationship between HOMA-IR and POR in patients without PCOS. Li et al. found a negative relationship between HOMA-IR and ovarian response in infertile patients with good ovarian reserve (16). The mechanisms underlying the declining ovarian sensitivity during COS are not fully understood. Insulin might play a crucial role in ovarian function and could be involved in promoting follicle development (17). In addition, previous research revealed that being overweight may suppress the ovarian response (18) and decrease the number of retrieved oocytes (19, 20). Increased insulin levels in obese sufferers will promote the generation of androgen in the ovary, leading to increased estrogen and creating a negative impact on the hypothalamic–pituitary–ovarian axis. Consequently, this can inhibit follicle growth by suppressing the formation of FSH and prolonging the duration of ovarian stimulation (21, 22). Fortunately, insulin resistance and body weight are reversible parameters. Due to the higher risk of POR, insulin and weight management should be encouraged to potentially improve the outcomes of assisted reproductive techniques (ART).

An appropriate COS protocol and an adequate starting dose of Gn are important to avoid unforeseen POR (23). Consistent with other research, it was revealed that the GnRH antagonist protocol and the initial dose of Gn are critical factors in estimating the chances of unexpected POR. A randomized clinical trial (RCT) uncovered a decrease in the average quantity of retrieved oocytes in the GnRH antagonist protocol (24). Wang et al. reported that the initial dose of Gn, FSH, and the LH level on the initial day of Gn administration were variables independently contributing to fewer oocytes retrieved than expected (25). A cohort study revealed that the COS protocol, average Gn dose, previous instances of POR, and a history of ovarian surgery were independent factors influencing the occurrence of POR (26). Some scholars have suggested that insufficient Gn is the main factor for suboptimal ovarian response in a long-agonist regimen (27). However, definitive criteria for selecting the optimal Gn starting dose have yet to be completely identified (9). Some researchers are developing indices to calculate the suitable initial exogenous dose of Gn. For instance, a nomogram was created by La Marca et al. to indicate the ideal initial FSH dose according to the age, AMH, and FSH levels of patients (28).

This study has some limitations that need to be highlighted. First, the inherent biases associated with retrospective analysis may impact the results. To mitigate selection bias, we set relatively broad criteria for ovarian reserve, BMI, and the COS protocol. We also excluded individuals with factors including untreated metabolic or endocrine abnormalities, pathological ovarian cysts, or history of ovarian surgery that could potentially influence the ovarian response. These inclusion and exclusion criteria were employed to ensure population homogeneity and to enhance practical clinical applicability. Moreover, we conducted model validation in non-PCOS patients to address the potential confounding of PCOS. Furthermore, limited by the data collection, information regarding weight loss during ovulation induction, the metformin dose in patients with insulin resistance, and posttreatment HOMA-IR levels remains unclear. However, our center adopted a standardized treatment for patients with insulin resistance and obesity without any bias across different populations. Therefore, further exploration is warranted to investigate relevant indicators and prediction models.

## 5 Conclusion

In summary, in this study, a predictive model was developed incorporating BMI, bFSH, AMH, AFC, HOMA-IR, protocol, and the initial dose of Gn as indicators to estimate the probability of POR in patients with good ovarian reserve. We anticipate that our model would be significant in evaluating unexpected POR occurrences and in providing assistance to clinicians in making clinical decisions.

## Data availability statement

The original contributions presented in the study are publicly available. Further inquiries can be directed to the corresponding author.

## Ethics statement

The studies involving humans were approved by Henan Provincial People’s Hospital Ethics Committee. The studies were conducted in accordance with the local legislation and institutional requirements. The ethics committee/institutional review board waived the requirement of written informed consent for participation from the participants or the participants’ legal guardians/next of kin because the retrospective nature of the research.

## Author contributions

XX: Writing – original draft, Conceptualization, Methodology, Writing – review & editing. XW: Conceptualization, Formal analysis, Methodology, Writing – original draft. YJ: Formal analysis, Software. HS: Formal analysis, Software. YC: Data curation. CZ: Funding acquisition, Project administration, Supervision, Writing – review & editing.
